# Examination of Cognitive Fatigue in Multiple Sclerosis using Functional Magnetic Resonance Imaging and Diffusion Tensor Imaging

**DOI:** 10.1371/journal.pone.0078811

**Published:** 2013-11-01

**Authors:** Helen M. Genova, Venkateswaran Rajagopalan, John DeLuca, Abhijit Das, Allison Binder, Aparna Arjunan, Nancy Chiaravalloti, Glenn Wylie

**Affiliations:** 1 Neuropsychology and Neuroscience Laboratory, Kessler Foundation Research Center, West Orange, New Jersey, United States of America; 2 Department of Physical Medicine and Rehabilitation, Rutgers, New Jersey Medical School, Newark, New Jersey, United States of America; 3 Department of Psychology, University of Massachusetts, Amherst, Amherst, Massachusetts, United States of America; 4 Department of Clinical Psychology, Suffolk University, Boston, Massachusetts, United States of America; University of Jaén, Spain

## Abstract

The present study investigated the neural correlates of cognitive fatigue in Multiple Sclerosis (MS), looking specifically at the relationship between self-reported fatigue and objective measures of cognitive fatigue. In Experiment 1, functional magnetic resonance imaging (fMRI) was used to examine where in the brain BOLD activity covaried with “state” fatigue, assessed during performance of a task designed to induce cognitive fatigue while in the scanner. In Experiment 2, diffusion tensor imaging (DTI) was used to examine where in the brain white matter damage correlated with increased “trait” fatigue in individuals with MS, assessed by the Fatigue Severity Scale (FSS) completed outside the scanning session. During the cognitively fatiguing task, the MS group had increased brain activity associated with fatigue in the caudate as compared with HCs. DTI findings revealed that reduced fractional anisotropy in the anterior internal capsule was associated with increased self-reported fatigue on the FSS. Results are discussed in terms of identifying a “fatigue-network” in MS.

## Introduction

Multiple Sclerosis (MS) is a disease of the Central Nervous System characterized by chronic inflammatory demyelination and both white and grey matter pathology. Individuals with MS suffer from a range of physical, psychiatric and cognitive symptoms, with fatigue being one of the most common (reported in 70-90% of patients) [1,2,3]. The Multiple Sclerosis Council for Clinical Practice Guidelines [4] has defined fatigue as “a subjective lack of physical and/or mental energy that is perceived by the individual or caregiver to interfere with usual and desired activities” and 40% of patients rate it to be their most troubling symptom. Yet, despite its prevalence and the negative impact it has on the lives of individuals with MS, researchers have had significant difficulty in studying and isolating fatigue. 

A consistent relationship between subjects’ self report of fatigue and putative objective measurements of fatigue, such as performance on a demanding task, has been elusive. The typical methodology in studies of objective measurements of cognitive fatigue, has consisted of measuring behavioral performance before and after inducing cognitive fatigue, either over a prolonged period of time (i.e. over the course of a work day or long neuropsychological battery) or during sustained mental effort (i.e. examining behavioral performance during the course of a neuropsychological task requiring constant cognitive effort). Generally, it has been hypothesized that as a subject’s self-report of fatigue increases, behavioral performance should worsen (see for review [5]). The overwhelming majority of studies have failed to show a consistent effect of prolonged cognitive fatigue on behavioral performance (i.e. subjects’ perception of increased fatigue does not relate to behavioral performance) [6,7,8]. Although studies which utilize the sustained mental effort approach have been more sensitive to cognitive fatigue, studies have been unable to consistently relate subjective measures of cognitive fatigue to objective indices such as behavioral performance (for review see [5]).

It has been argued that subjective and objective measures of fatigue fail to consistently correlate because behavioral performance is not the best measure of cognitive fatigue [5,9]. Specifically, individuals with MS may be able to perform a task at normal levels (comparable to HCs) but their increased perception of fatigue may be reflective of increased “cerebral work.” (i.e., allocation of more cerebral resources to perform at a similar level). Support for increased cerebral activity associated with objective cognitive fatigue was shown by DeLuca et al. [10] using fMRI. This study noted that across performance of a sustained cognitive task, increased activation in the MS group relative to healthy controls was observed in the basal ganglia and frontal and parietal areas. Findings were replicated by this same group in persons with traumatic brain injury [11]. These findings are consistent with the model of central or cognitive fatigue proposed by Chaudhuri & Behan [12]. In this model, it is hypothesized that central fatigue is associated with dysfunction of the non-motor functions within the basal ganglia which in turn negatively impacts the striatal-thalamic-frontal cortical system [12].

While DeLuca et al. [10] and Kohl et al. [11] were able to use fMRI as an objective measure of fatigue, they did not include self-report fatigue measures to examine the relationship between self-reported fatigue and patterns of cerebral activation. The present study was designed to address this limitation of previous research. Specifically, the current study examines the neural correlates of self-reported “state” cognitive fatigue using fMRI (Experiment 1). “State” fatigue refers to a transient condition, which can change with time, and can fluctuate based on both internal and external factors. The examination of “state” fatigue has been largely neglected in previous research, as most studies have examined “trait” fatigue (i.e. [13,14]). In experiment 1, we hypothesized that self-reported fatigue assessed during task performance (i.e., via a Visual Analog Scale of Fatigue, see methods) would increase significantly during a sustained cognitive challenge and would be associated with increased brain activity in a network of regions involved in cognitive fatigue. Specifically, we examined brain activity in the basal ganglia, frontal lobes and thalamus given the role of the striatal-thalamic-frontal cortical system in fatigue as proposed in the model by Chaudhuri & Behan [12].

Further, we also examined the neural correlates of “trait” fatigue (Experiment 2). “Trait” fatigue refers to a more stable state in an individual, and is not likely to change significantly over time. Experiment 2 utilized diffusion tensor imaging (DTI) to examine the relationship between white matter integrity and cognitive ‘trait’ fatigue in a separate sample of individuals with MS. For this analysis, we investigated the relationship between white matter integrity and a self-report measure of fatigue that assesses the experience of fatigue over the past week (the Fatigue Severity Scale [15]). We hypothesized that reduced integrity of white matter tracts would be associated with increased “trait” fatigue in the MS sample. Specifically, we predicted a relationship between increased subjective “trait” fatigue and damage to white matter tracts throughout the striatal-thalamic-frontal cortical system, based on the model proposed by Chaudhuri & Behan [12,16].

## Methods

Institutional review boards responsible for ethical standards at UMDNJ and the Kessler Foundation approved both studies. Written informed consent was obtained from all participants prior to participation. All methods used for data collection were HIPAA compliant. 

### Experiment 1: Methods

#### Participants

 Experiment 1 included 23 right-handed participants: 11 healthy controls (8 females) and 12 individuals with MS (10 females). All MS participants were diagnosed with clinically definite MS [17]. Nine of the MS participants were classified with relapsing-remitting MS, one with primary-progressive MS, and one with secondary progressive MS [17]. All participants had normal or corrected-to-normal visual acuity and normal color vision. Participants with a history of diagnosed psychological and psychiatric problems (i.e. resulting in patient hospitalization for these disorders), epilepsy, learning disability, diagnosis of substance abuse/dependence, brain injury or episodes of loss of consciousness (lasting 30 or more minutes) were excluded. We excluded any participant with MS who experienced an exacerbation within a month prior to testing or were taking corticosteroids. In the MS group, the average time since diagnosis was 12.1 years (± 11.97 SD). Years of education did not differ significantly between the HC (M = 16.2, SD =1.1 years) and MS (M = 14.9, SD = 2.1 years) groups (M = -1.265, 95% CI [-2.715, .184], t(16.878) = -1.863, p = .080). According to Levene’s test for equality of variance, homogeneity of variance could not be assumed for the t-test comparing ages between groups (p = .007), so separate variances and the Welch-Satterthwaite correction were used. The MS group was significantly older (46.8 ± 7.1 years) than the HC group (32.3 ± 11.8 years), (t(21) = 3.61, p =.002). 

#### Neuropsychological Testing

All participants completed a battery of neuropsychological tests targeting areas of cognitive function commonly impaired in individuals with MS. Executive functioning and processing speed were measured through several subtests of the Delis-Kaplan Executive Function System [18] including the Trail-Making Test, the Color-Word Interference Test, and Tower Task. These tests examine specific components of executive abilities such as mental flexibility, planning, problems solving, impulsivity, and inhibition, and rely on processing speed and executive ability. Premorbid IQ was assessed using the Reading Subtest (WRAT-3; [19]) and Vocabulary and Block Design subtest of the Wechsler Abbreviated Scale of Intelligence (WASI; [20]). 

#### Apparatus

A Siemens 3T ALLEGRA magnet was used for both the functional and anatomical data collection. Head motion was minimized using the standard Siemens head-holder. Stimuli were delivered using a back-projection system: the stimuli were presented onto a screen mounted on the end of the magnet bore above the subject’s head, and a mirror was mounted on the head coil – directly in the subject’s line of sight – that allowed him/her to see the screen. 

The generation and sequencing of stimuli and the collection of subject responses were accomplished using the Presentation® software package (Version 0.75, http://www.neurobs.com/). This was run on a laptop that was interfaced with the back-projection system. Responses were collected using an MR compatible, two-button response pad (Lumina LP-400 response pad system; Cedrus®, http://www.cedrus.com/). All participants responded with the index and middle fingers of their right hand.

#### Task Switching Paradigm

**Figure 1:**
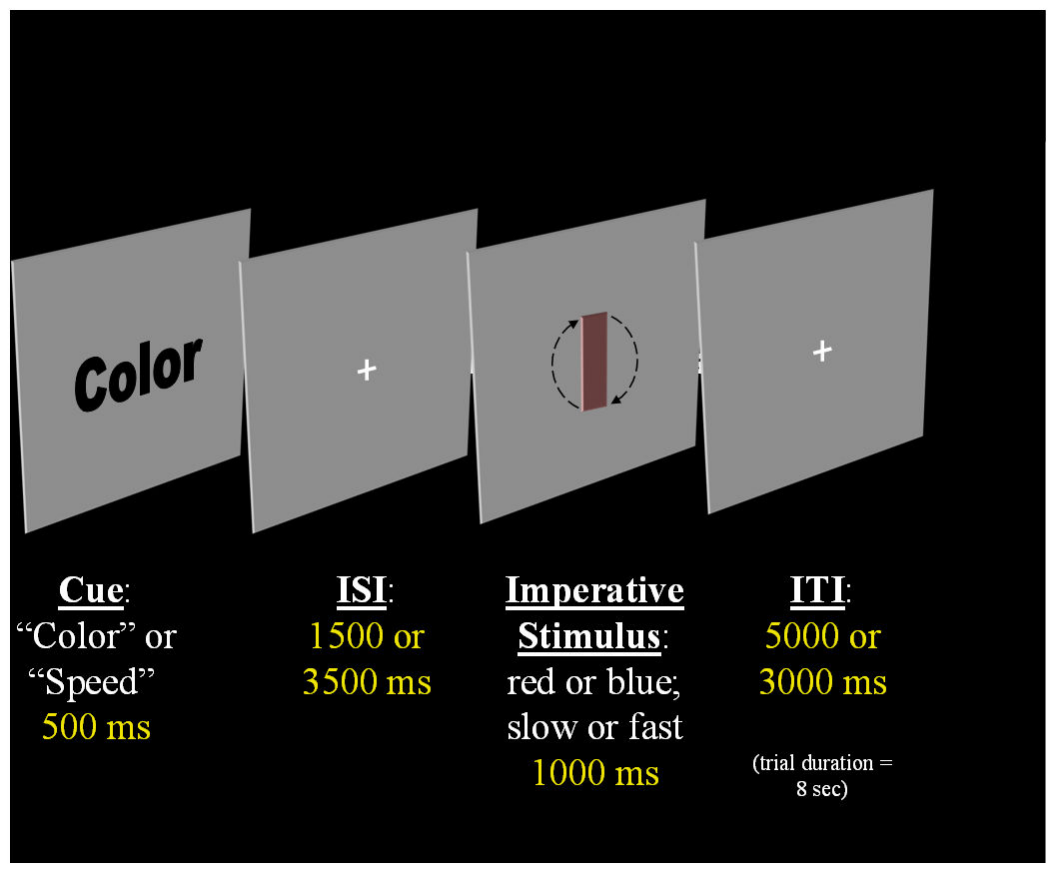
Task Switching Paradigm. Figure 1. shows an example of the task-switching paradigm performed in Experiment 1.

We modified an experimental procedure that required participants to switch between two sets of tasks [21] to induce cognitive fatigue in the scanner. Examples of the stimuli can be seen in Figure 1, where the sequences of events comprising a single trial are depicted. Participants switched randomly between two tasks – a color judgment task and a speed judgment task – based on a cue that was presented on every trial. (In order to avoid confounding task-switching from cue-switching [22], two cues were used for each task: ‘color’ and ‘hue’ were used to cue the color judgment task; ‘speed’ and ‘velocity’ were used to cue the speed judgment task.) 

The imperative stimuli were colored, rotating rectangles. In all cases, the rectangles were delimited by a black outline. The rectangles were tinted either red or blue. The red, green, blue (RGB) values (range = 0–254) were [128,100,100] or blue (RGB = [100,100,128]), and rotated either quickly (100 frames per second [f/s] at 10° per frame) or slowly (40 f/s at 10° per frame). Additionally, a low-level baseline condition was included. This was an ‘empty’ trial. The cue presented was the word ‘Empty’, and no imperative stimulus was presented on these trials. Participants were told to expect these trials and to simply wait until the next cue was presented. 

Three sequences of stimuli were generated using the RFSgen program of the AFNI suite [23]. This allowed us to ensure that the sequences would be amenable to deconvolution before the data were collected. Each subject was run using one of these three sequences, and the sequences were counterbalanced across participants. In total, 192 trials were run, divided into 6 blocks (32 trials per block). 

On each trial, a cue was presented for 500 ms, followed by a cue-to-target interval (CTI) that was varied between 1500 and 3500ms. Both of these CTIs are sufficiently long to allow participants to achieve maximal preparation for the forthcoming task [24]. However, varying the CTI in this way has been shown to allow for later deconvolution of the hemodynamic response associated with the cue [25]. The task stimulus was then presented for 1000 ms, followed by an inter-trial interval. In all cases, each trial lasted a total of 8 s. 

#### Procedure

All participants were familiarized with the tasks and the procedure prior to entering the scanner, and worked through one practice block (comprised of 32 trials, containing at least one instance of each of the conditions) to ensure complete understanding of the tasks. Instructions were to respond as quickly and accurately as possible, and to maintain fixation throughout each trial. Performance was monitored, and participants were required to work through additional practice blocks if it appeared that they had not understood the instructions. 

Employing an event-related paradigm, subjects worked through six experimental runs while in the scanner, each of which was comprised of 32 trials. Each of the 6 runs began with an instruction screen that reminded participants of both the cues that were associated with each task and the response assignments for each task. This was presented for 7 s. Then a countdown from 3 to 1 was presented, with each number presented for 1 s. This initial 10 s (5 TRs), allowed the magnetic field to achieve a steady state. Following this period, the experimental trials were presented, and each run lasted ^~^4.5 min. 

#### Measuring “state” fatigue

A visual analogue scale (VAS) was used to measure “state” cognitive fatigue during the demanding task switching paradigm while in the scanner. The VAS-F has been established as a valid and reliable instrument for the measurement of self-reported fatigue [26,27]. Before and after each of the six runs of the cognitive task, subjects were asked to report on a scale of 0-100, how mentally fatigued they felt “right now, at this moment.” Unlike other fatigue scales, where subjects are asked to estimate their fatigue over an extended period of time (i.e. the previous week), we asked subjects to focus on their feelings of fatigue at that moment, without regard to previous feelings of fatigue, making our implementation of the VAS a better measure of “state” fatigue. In addition, to separate self reported fatigue from other self reported feelings, subjects were also asked to rate their happiness, sadness, tension, anger and pain, each using the same VAS 0-100 scale. This was done because self-reported fatigue is often associated with self-reported emotional distress, so we included these ratings in the deconvolution (see below) – thus ensuring that any activation we found was specific to self reported fatigue.

#### fMRI Data Collection

Information about the hemodynamic response evoked by the tasks was obtained using single-shot, T2*-weighted, echo planar imaging sequences on the Siemens 3 T. Images were acquired with a TR of 2 s, a TE of 30 ms and a 80° flip-angle. Each of the volumes consisted of 32 slices (voxel size = 3. 438 × 3. 438 × 4 mm; matrix size = 64 × 64 voxels), which allowed for whole-brain coverage. In each of the six experimental blocks, 135 volumes were acquired. Prior to data analysis, the first five volumes of each block were discarded to account for the time needed for the field to achieve a steady state. 

#### Anatomical MRI

High-resolution whole brain images were acquired using the Siemens 3 T magnet with a 3D T1-weighted magnetization-prepared rapid gradient echo sequence. A total of 144 slices were acquired (voxel size = 0.859 x 0.859 mm; slice thickness = 1 mm; matrix size = 256 × 256; TE = 4.38 ms; flip-angle = 8°). These anatomical images were acquired for co-registration with the fMRI data and for analyses of voxel-based morphometry (VBM). For lesion quantification, A FLAIR sequence was acquired (3D inversion-recovery sequence acquired axially; TR = 8530 ms, TE = 81 ms, flip angle = 180 deg, 256 x 256 matrix, FOV = 220 mm, NEX = 1, 32 slices, 4 mm slice thickness, 0 mm skip).

#### Lesion Load Analysis

The MS subjects’ FLAIR-weighted images were used to estimate lesion load volume. The Lesion Segmentation toolbox in SPM8 was used for this quantification [28]. The processing steps include: 1) the FLAIR weighted images were bias corrected, 2) tissue class maps of grey and white matter, and cerebrospinal fluid were obtained using the T1-weighted images, 3) FLAIR intensity distribution of each tissue class was obtained where the outliers correspond to lesions which are interpreted as lesion belief maps, 4) a liberal lesion belief map was obtained and neighboring voxels were iteratively assigned to lesions. Total lesion volume for each subject was measured using the probability lesion map obtained from previous step.

#### Voxel Based Morphometry (VBM)

Grey matter VBM analysis was carried out using openware FSL version 5.0 (http://www.fmrib.ox.ac.uk/fsl/) [29] adopting the standard VBM pipeline. T1-weighted images were corrected for lesions using Lesion segmentation toolbox in SPM8 [28] and transferred to FSL for VBM analysis. An optimized VBM approach, developed by Good and colleagues, was adopted [30,31]. Data processing was divided into four major steps: 1) T1-weighted images were brain-extracted using BET [32] adopting the methods of Popescu et al. [33]. 2) Brain extracted images were segmented into WM, GM and cerebrospinal fluid (CSF) volume probability maps using FAST [34]. 3) a study-specific GM template was created. This was done by registering the 11 HCs and 11 of the 12 MS subjects (randomly chosen to avoid bias during the registration process) into the MNI152 space using the affine registration tool FLIRT [34,35]and then by nonlinear registration using FNIRT (www.fmrib.ox.ac.uk/analysis/techrep). The resulting images were averaged to create the template. 4) All the native GM images were non-linearly re-registered to the template and modulated using the Jacobian of the warp field. 5) These images were then smoothed using a full-width half-maximum (FWHM) of 7 mm, and 6) general linear model (GLM) was used to compare voxel-wise differences in GM volume between the MS and HC groups after regressing out age. Non-parametric statistics was performed using “randomise” with 5000 permutations and using threshold free cluster enhancement (TFCE) option. A p<0.05 corrected for multiple comparisons using family wise error rate (FWER) was used as the level of significance.

#### fMRI Data Analyses: state fatigue

All images were realigned using the AFNI suite of image analysis tools [23] and any blocks in which a subject moved more than one voxel in any dimension or more than a degree in pitch, roll or yaw were discarded (none of the data from any subject fulfilled these criteria). Each raw time-series of signal strength for each subject was first time-shifted so that the slices were ‘aligned’ temporally (i.e. shifted so that the slices have the same temporal origin), and any linear trends in the data were removed. All of the volumes in the time-series were then spatially registered using an image midway through the time-series as the canonical image. All voxels outside the brain were eliminated from further analysis. The hemodynamic response was modeled by a delayed gamma function, and this function was coded into the design matrix as a regressor. Contrasts were specified using the General Linear Model (GLM). 

To assess which areas in the brain showed functional changes in activity that correlated with ratings of subjective state fatigue (on the VAS-F), each trial (in the event-related design) was modeled with seven regressors. The first was simply a delayed Gamma function with unit amplitude. This regressor captured the brain response that was invariant across trials/events (e.g., basic visual processing). For the purpose of investigating cognitive fatigue, this regressor was not of interest. The six remaining regressors were designed to capture the brain response specifically associated with each subjective rating (“state” cognitive fatigue, pain, sadness, happiness, tension, anger). These regressors were also delayed Gamma functions, but the amplitude of this function for each event depended on each subject’s subjective report of each state. That is, these six regressors were Amplitude Modulated Regressors (AMRs). In the analyses below, we focused exclusively on the fatigue AMR.

The amplitude of the fatigue AMR for each event was determined by linearly interpolating the scores provided before and after each of the six fMRI runs. Thus, if a subject rated his/her “state” cognitive fatigue as 0 before a given run and 40 following that run, an event that occurred halfway through the run would have an amplitude of 20 (or 0.20, since all events were scaled to vary between 0 and 1). An event that occurred three-quarters of the way through the run would have an amplitude of 0.30, and so on. 

Images were thresholded to protect against type I error following Baudewig, Dechent, Merboldt & Frahm (2003) [36]. All results were significant at a corrected alpha level of p < .05 (i.e. results were corrected for multiple comparisons). The correction was done using a Monte Carlo simulation showing that with an individual probability threshold of p < .01, clusters of at least 66 contiguous voxels (in native space) resulted in a corrected alpha of p < .05. When we constrained our analyses to specific Regions of Interest (ROIs) (i.e. basal ganglia, thalamus and frontal lobes), we found that with an individual probability threshold of p < .01, the Monte Carlo simulation showed that clusters of at least 33 contiguous voxels resulted in a corrected alpha of p < .05.

## Results

### Experiment 1: Results

#### Behavioral Results: “State” Fatigue

Data analyses were conducted using SPSS statistical computing environment. Independent samples t-tests were run to compare performance between the MS and HC groups. Neuropsychological test results are presented in Table 1. Because age was significantly different between groups, age-corrected standard scores were used to correct for group differences in age. There were no significant differences between the MS group and HCs on any of these tests.

**Table 1:** Experiment 1: Means, Standard Deviations, t-values for performance on neuropsychological tests.

	**MS**	**HC**	**t**	**p**
**Test**	*Mean (SD)*	*Mean (SD)*		
*WRAT reading*	102.25 (10.52)	108.18 (5.19)	-1.69	0.11
*WASI Vocab*	12.25 (1.66)	11.73 (1.68)	0.75	0.46
*WASI Blocks*	11.92 (2.35)	11.82 (2.32)	0.1	0.92
*DKEFS Trails: Number Sequencing*	11.33 (1.88)	12.18 (2.32)	-0.97	0.34
*DKEFS Trails: Letters Sequencing*	11.67 (1.97)	12.18 (1.6)	-0.68	0.5
*DKEFS Trails: Number Letter Switching*	11.51 (1.73)	11.55 (1.37)	-0.07	0.95
*DKEFS Color Word Interference: Color Naming*	9.17 (3.1)	9.09 (1.87)	0.07	0.95
*DKEFS Color Word Interference: Word Reading*	10.33 (3.68)	10.09 (2.12)	0.12	0.85
*DKEFS Color Word Interference: Inhibition*	10.67 (3.34)	11.64 (1.5)	-0.88	0.39
*DKEFS Tower Task: Total Achievement*	11.17 (2.41)	11.18 (2.96)	-0.01	0.99

Note: for all Neuropsychological tests, scaled scores were used.

The groups were additionally compared in terms of response accuracy and reaction time for the fMRI task-switching paradigm using ANCOVAs with age added as a covariate. Data for 4 subjects was not available for analysis due to acquisition equipment failure. No group differences were observed for either accuracy (F (1,16)=1.99, p=.178) or reaction time (F(1,16)=.032). 

**Figure 2:**
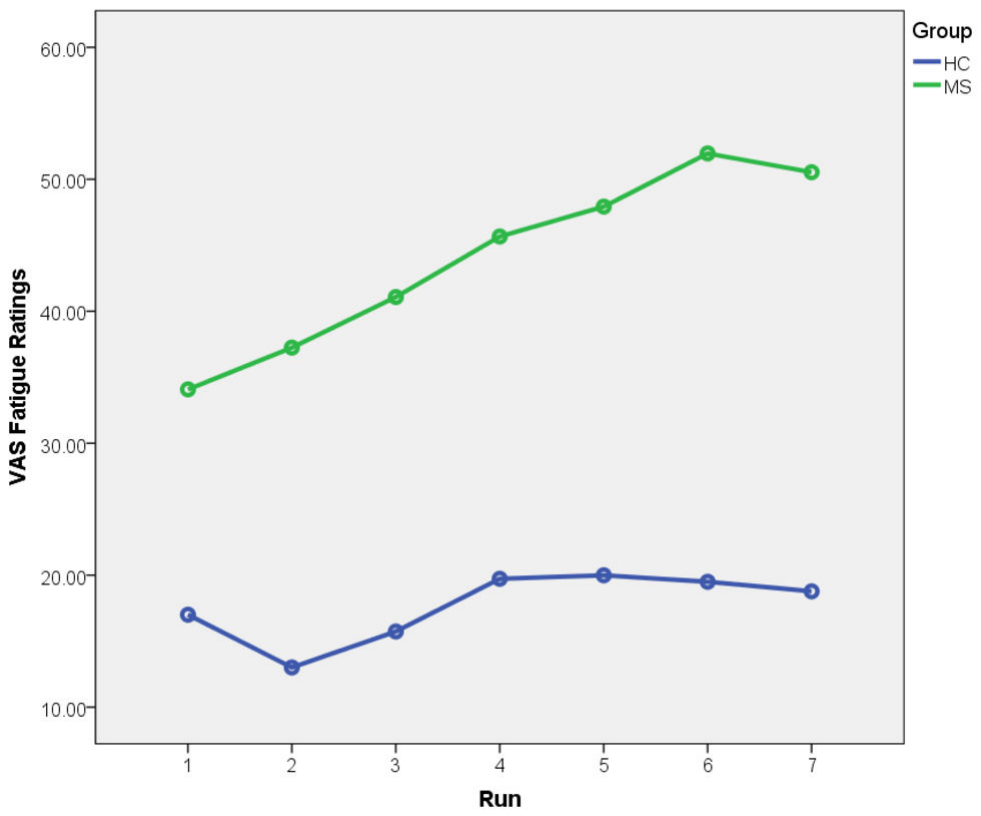
Fatigue across run for MS and HC group. Experiment 1: Results of group (2) x run (7) mixed design ANCOVA (age as covariate) examining self-reported VAS ratings of fatigue across 7 runs of fMRI cognitive task. MS group reported more fatigue relative to the HC group but there was no significant group x time interaction.

The VAS fatigue ratings were analyzed using a 2 x 7 mixed design ANCOVA, with group (MS vs. HC) as the between-groups factor and run (runs 1 to 6; NB, there are seven levels to this factor because ratings were acquired before and after each of the six runs) as the within-subjects factor, age was added as a covariate. Mauchly's test of Sphericity showed that the sphericity assumption did not hold and adjusted degrees of freedom were therefore used (Greenhouse Geisser correction). There was no significant main effect of run (F(2.82, 56.41)=.823). The main effect of group was significant (F(1,20)=6.52, p=.019) with the MS group reporting more fatigue relative to the HC group (see Figure 2). However, the group by time interaction was not significant (F(2.82, 56.41)=.754). 

#### Lesion Load and Voxel Based Morphometry

Mean lesion load was 9.74 + 11.86mm for the MS subjects. Regarding differences in GM volume (based on the VBM analysis), no significant GM volume changes were observed between the groups.

#### Neural Correlates of ‘State’ fatigue

In order to assess which areas were associated with self-reported ‘state’ cognitive fatigue (assessed with VAS scores), the activation associated with the fatigue AMR was examined in each group. Additionally, activation associated with fatigue AMR was compared between groups (MS vs. HC) using a t-test. The BOLD activation of each group is detailed in Table 2. Self-reported “state” cognitive fatigue was associated with increased activation in the MS group in bilateral prefrontal cortex (in clusters which included middle frontal gyrus, insula and putamen/caudate), left postcentral gyrus, precuneus, precentral gyrus (in a cluster of voxels which also included right inferior frontal gyrus and insula) inferior temporal gyrus, and declive of the cerebellum, but associated with negative activation in the left superior frontal gyrus, right cuneus and bilateral temporal regions. In the HC group, activation was positively associated with self-reported “state” cognitive fatigue in the following regions: left insula, left precentral gyrus, right thalamus/substantia nigra, right middle occipital gyrus, and right cumen, but negatively associated with activation in right medial frontal gyrus, left posterior cingulate, left superior occipital gyrus, and left middle temporal gyrus. When examining whole brain differences between HCs and the MS group, no differences were found.

**Table 2:** BOLD activation during performance of cognitively fatiguing task, associated with self-reported state fatigue (VAS-Fatigue).

	**Group**	**BA**	**Voxels**	**X**	**Y**	**Z**	**t-value**
***PositivelyAssociatedwithFatigue***							
**Frontal**							
**Left Insula**	**HC**	13	87	-31	19	11	4.43
**Left Middle Frontal Gyrus**	**MS**	6	470	-52	4	38	3.18
*Insula*		*13*	*-*	*-34*	*17*	*9*	*5.08*
*Putamen/Caudate*			*-*	*-17*	*11*	*4*	*6.48*
**Fronto-Central**							
**Left Precentral Gyrus**	**HC**	4	894	-34	-25	68	3.89
**Left Postcentral Gyrus**	**MS**	1&3	1566	-37	-31	65	3.32
**Right Precentral Gyrus**	**MS**	6	406	40	-13	59	3.87
*Right Inferior Frontal Gyrus*		*9*	-	*42*	*4*	*28*	*4.56*
*Insula*		*13*	-	*35*	*18*	*6*	*6.48*
**Parietal**							
**Right Precuneus**	**MS**	39	110	31	-61	38	3.35
**Temporal / Occipital**							
**Right Middle Occipital Gyrus**	**HC**	18	93	22	-94	14	3.81
**Right Inferior Temporal Gyrus**	**MS**	37	158	43	-67	0	3.48
**Cerebellar**							
**Right Cumen**	**HC**		177	25	-46	-27	5.85
**Right Declive**	**MS**	37	223	40	-55	-21	3.45
**Thalamic / Striatal**							
**Right Thalamus and Right Substania Nigra**	**HC**		142	7	-13	-6	3.40
***NegativelyAssociatedwithFatigue***							
**Frontal**							
**Right Medial Frontal Gyrus**	**HC**	10	293	1	55	5	-3.49
**Left Superior Frontal Gyrus**	**MS**	9	1151	-25	58	35	-4.59
**Parietal**							
**Left Posterior Cingulate**	**HC**	30,31	110	-1	-52	23	-4.33
**Right Cuneus**	**MS**	19	749	1	-82	38	-4.31
**Occipital / Temporal**							
**Left Superior Occipital Gyrus**	**HC**	19	131	-34	-85	29	-3.53
**Left Superior Temporal Gyrus**	**MS**	22	66	-58	4	-3	-3.59
**Left Middle Temporal Gyrus**	**MS**	39	182	-52	-70	26	-4.0
**Left Middle Temporal Gyrus**	**HC**	21	122	-64	-25	0	-4.84
**Right Middle Temporal Gyrus**	**MS**	39	103	46	-73	26	-3.25

The results are organized to show similarity in activation across groups. ‘BA’ denotes the Broddman Area where the voxel with the peak activation was located. ‘Voxels’ denotes the number of voxels in a cluster. The location of the voxel with the peak activation is denoted by X, Y and Z coordinate. ‘t-value’ denotes the t-statistic at the voxel with the peak activation. Italicized rows denote sub-regions in the larger cluster.

**Figure 3:**
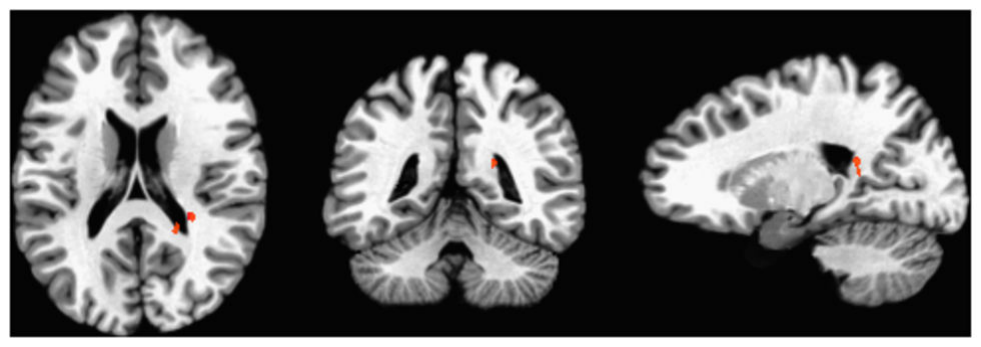
Caudate activity greater in MS group relative to HCs. Figure 3. shows activation in the caudate which was greater in the MS group compared to HCs.

Because of the role of the striatal-thalamic-frontal cortical system in fatigue [10,12], we performed ROI analyses on the basal ganglia, thalamus and frontal lobes, in which we compared the fatigue-related activation across the two groups (i.e., MS – HC). These analyses revealed a cluster in the caudate (See Figure 3; caudate tail: 20, -42, 16) that was significantly more active in the MS group (p < .01,) compared to HCs. 

### Experiment 1: Discussion

During performance of the task-switching paradigm, both groups showed brain activity positively associated with “state” fatigue in prefrontal regions and cerebellum, but negatively associated with “state” fatigue in temporal regions. These regions have been implicated in fatigue in HC [37], as well as in other clinical populations such as Chronic Fatigue Syndrome [38]. The pre and post central gyrus as well as the temporal lobes are highly connected to the striatum, and therefore these brain regions are consistent with the striatal-thalamic-frontal cortical system’s implicated role in fatigue [12,16]. When comparing the two groups, more activation was observed in the caudate in the MS group compared to HCs. Our findings therefore indicate that the MS group required increased activation of the caudate relative to HCs in response to increasing “state” fatigue. 

### Experiment 2: Methods

#### Participants

Experiment 2 included 25 right-handed participants: 12 HCs (7 female) and 13 individuals with MS (12 female). All MS participants were diagnosed with clinically definite MS [17]. Of these 13 individuals, 12 were female and 11 had relapsing-remitting MS, while the remaining 2 had secondary-progressive MS. All participants had normal or corrected-to-normal visual acuity and normal color vision. Participants with a history of diagnosed psychological and psychiatric problems (i.e. resulting in patient hospitalization for these disorders), epilepsy, learning disability, diagnosis of substance abuse/dependence, brain injury or episodes of loss of consciousness (lasting 30 or more minutes) were excluded. As in Experiment 1, we excluded any participant with MS who experienced an exacerbation within a month prior to testing or were taking corticosteroids. The mean number of months since diagnosis for the MS group was 185.7 ± 114.4 months. There was no significant difference in terms of education (t (23) = -.822, p = .42) between the MS group (M = 16.7 years, SD = 2.2) and HC group (M = 15.9, SD = 2.5). The MS group was significantly older than the HCs (t (23) = -2.78, p = .01): MS group had a mean age of 47.8 years (SD = 11.0 years) while the HCs had a mean age of 36.4 years (SD = 9.2). Due to the significant difference in age between the groups and the known impact of age on white matter integrity (i.e. [39]), age was used as a covariate in all subsequent analyses.

#### “Trait” Fatigue questionnaire

We used the Fatigue Severity Scale (FSS) to measure “trait” fatigue in the participants. This test was administered immediately prior to the scanning session. The FSS is a 9-item scale used to assess fatigue in various clinical disorders, including MS [15] . Each of the nine items is completed on a rating scale of 1 to 7, and a final score is averaged from the total of the nine ratings. The FSS has been validated in a variety of clinical, behavioral and neuroimaging studies to investigate fatigue and specifically, fatigue in MS [40,41,42,43,44]. Because subjects are asked to rate the fatigue they have experienced over an extended period of time (the previous week), the FSS is a highly appropriate tool for the assessment of trait fatigue. Indeed some have used it to estimate fatigue for time periods as long as 6 months [45].

#### Anatomical data collection

Patients were scanned on a 3T Siemens Allegra System. Scans included an MPRAGE and a T2-weighted image. For lesion quantification, A FLAIR sequence was acquired (3D inversion-recovery sequence acquired axially; TR = 8530 ms, TE = 81 ms, flip angle = 180 deg, 256 x 256 matrix, FOV = 220 mm, NEX = 1, 32 slices, 4 mm slice thickness, 0 mm skip). DTI data were acquired using a 12-directional echoplanar sequence (TR=7300ms, TE=88ms, FOV=210mm, matrix=128x128, slice thickness 4mm, 26 slices, no gap, b=1000 s/mm^2^, NEX=7). After acquisition, data were transferred off-line to a Linux-based workstation and processed using FSL from the FMRIB software library (Functional Magnetic Resonance Imaging of the Brain Software Library; http://www.fmrib.ox.au.uk/fsl). For each participant, a single FA image was created from the raw diffusion data as follows. First, DICOM (Digital Imaging and Communications in Medicine) ﬁles of each DTI acquisition were converted into a single multivolume NIfTI (Neuroimaging Informatics Technology Initiative) ﬁle. FSL’s Brain Extraction Tool (BET) was used to remove all non-brain tissue from each image by creating a brain mask from the B=0 (nondiffusion weighted) images. Each subject’s data were then corrected for distortions due to eddy currents and head motion using affine transformations. Finally, using DTIFIT from FSL, a diffusion tensor model was fit at each voxel, resulting in a single FA image. 

After the preprocessing steps had been completed, a white-matter “skeleton” was created using scripts within the Tract-Based Spatial Statistics (TBSS) package in FSL 4.1.4 [46]. The TBSS method minimizes potential misalignment problems of other voxel-based whole-brain analysis methods by first determining an FA skeleton restricted only to the center of major white-matter tracts. This skeleton was derived by running a non-linear registration that aligns all FA images to the FMRIB58_FA standard image supplied in FSL. FA values for each individual are then mapped directly onto the standard skeleton (MNI152 space) for group comparison [46]. 

An average lesion mask was obtained from all the MS patients. The lesion mask was used to exclude WM lesions from TBSS analysis [47]. Any data in the skeletonized images that overlapped with the lesion mask was excluded from further analysis. The lesion excluded skeleton images were then thresholded to protect against Type I error following Baudewig, Dechent, Merboldt, & Frahm [36]. All results were significant at a corrected alpha level of p < 0.05 (i.e., all results were corrected for multiple comparisons). The correction was done using a Monte Carlo simulation that showed that with an individual probability threshold of p<0.01, clusters of at least 38 contiguous voxels (in native space) were significant at p<0.05.

Lesion Load and grey matter VBM were processed and analyzed using identical methodology as that described in Experiment 1. 

#### Statistical Analysis

FA was analyzed using multiple linear regressions (the 3dRegAna program in the AFNI software suite) [23]. The analyses were run using the FSS as a fatigue assessment inventory, investigating its relationship with FA. The regression model included age and education as covariates as they are both variables known to affect white matter integrity (i.e. [39]). 

### Experiment 2: Results

#### Behavioral Results: “Trait” Fatigue

To compare fatigue levels between groups, an ANCOVA was used with age as a covariate (as age differed between groups). As expected, mean FSS scores were significantly higher in the MS group (FSS Mean = 51.8, S.D. = 11.5) compared to the HC group (FSS Mean = 17.8, S.D. = 7), (F (1,22) = 54.5, p < .001) after controlling for age.

#### Lesion Load Analysis and Voxel Based Morphometry

Mean lesion load was 8.13 + 12.28 mm for the MS subjects. There was no significant relationship between FSS scores and lesion load. Regarding differences in GM volume (based on the VBM analysis), no significant GM volume changes were observed between the groups, and there was no relationship between GM volume and FSS. 

#### DTI: “Trait” Fatigue

In the MS group, FSS scores were associated with reduced FA in only one 40 voxel cluster, the anterior internal capsule (R^2^=.427, p < .01). No significant relationship was noted between FA levels and FSS scores in the HC group.

### Experiment 2: Discussion

Findings from Experiment 2 showed that “trait” fatigue was associated with decreased white matter integrity in the anterior internal capsule in only the MS group (not observed in the HC group). The anterior internal capsule has connections through the caudate and thalamus, and therefore these findings further support the role of the striatal-thalamic-frontal system in fatigue. 

## Discussion

The major purpose of the current study was to examine the neural correlates of cognitive fatigue. This was accomplished through two experiments: the first examined the relationship between self-reported “state” fatigue and cerebral activation, while the second examined the relationship between self-reported “trait” fatigue and white matter integrity. The overall results of both experiments are consistent with the hypothesis that the striatum and its interconnections have a critical role in the subjective perception of fatigue in MS. 

In the first experiment, we found that “state” fatigue experienced while performing a task-switching task was associated with brain activity across several regions including prefrontal cortex, temporal lobes and the cerebellum in both HC and MS groups. Several of these regions (frontal) were observed to be related to fatigue in our previous study using a different task and different methodologies [10] while the other regions identified as important in cognitive fatigue in the current study (temporal lobe) have been found to be related to fatigue by others [37,38]. 

 When we examined what brain regions were more active in MS compared to HCs, we observed only one region: the caudate. This finding further supports the model presented by Chaudhuri and Behan [12,16], which indicates that disruption of the pathways through the basal ganglia via the associated loop of the striato-thalamo-cortical fibers may be primarily responsible for central fatigue. Further, the results of experiment 2 showed that lower FA in the anterior internal capsule was related to increased “trait” fatigue as assessed by the FSS in individuals with MS. The anterior internal capsule contains thalamocortical fibers which connect the medial and anterior nuclei of the thalamus to the frontal lobes. Therefore, the findings of both experiments, which used two different neuroimaging modalities (fMRI, DTI) and two independent samples to examine two types of fatigue (“state” and “trait”) converge to implicate a network of regions in fatigue: the basal ganglia, thalamus and frontal lobes. Taken together, these two studies provide strong support for the Chaudhuri & Behan [12] model of central fatigue.

The striatal-thalamic-frontal network has been hypothesized to underlie fatigue in other clinical populations. For example, it was recently observed that individuals with Parkinson’s Disease with significant fatigue had reduced serotonergic function in the basal ganglia compared to patients without fatigue [48]. Tang et al. [49], found that individuals with fatigue following stroke are more likely to have had infarcts in the basal ganglia, and specifically the caudate, compared to individuals without fatigue following stroke [50]. Researchers in our lab have observed cognitive fatigue to be associated with brain activity in the basal ganglia and frontal lobes using fMRI in individuals with moderate to severe TBI [11] and MS [10]. Finally, in MS, multiple studies have begun to link striato-thalamic-frontal regions to fatigue. For example, pathology/dysfunction of the striatum, thalamus, superior frontal gyrus and inferior parietal gyrus have been linked to increased self-reported fatigue in MS [13,51,52,53].

Interestingly, although differences were observed between HCs and the MS group in terms of levels of self-reported fatigue, and associated neural networks, no differences were noted between the groups in terms of neuropsychological functioning or in terms of performance on the fMRI task in experiment 1. Therefore, it appears that the fatigue-network identified in the current investigation is associated with increased perception of fatigue that is not necessarily associated with decreased performance on behavioral tasks. This lack of a relationship between self reported fatigue and behavioral performance is consistent with what has been observed for over 100 years in the behavioral literature [5]. Despite subjective reports of increased fatigue during performance of cognitive tasks or over time, there has been inconsistent relationship between what the subject reports, and their behavioral performance [6,7,8]. The findings of the current study, which indicate that brain activation increases with subjective reported fatigue without behavioral decrement in the MS group relative to HC, are consistent with the notion that behavioral performance is not the best objective measure of fatigue. Rather, researchers should begin to investigate neural networks of cognitive fatigue, which may be a more appropriate objective measurement of the subjective feelings of fatigue. 

The present study has several limitations. The current study was focused on the evaluation of cognitive or mental fatigue, while questions on the FSS are not specific to cognitive fatigue per se and likely reflect a more global assessment of the construct (e.g., (physical and cognitive fatigue). Despite the fact that various measures of subjective fatigue are highly correlated (e.g. [15] ), future studies might consider use of a measurement tool specific to cognitive fatigue when assessing subjective measures of “trait” cognitive fatigue. For example, the high scores on the Modified Fatigue Impact scale (MFIS [54]; have been shown to correlate with cortical atrophy of the parietal lobes [55]. The MFIS has a subscale assessing cognitive fatigue, and therefore maybe a more appropriate measure of “trait” cognitive fatigue than the FSS. Additionally, participants that took part in Experiment 1 were not the same as those in Experiment 2, making it difficult to directly relate the findings of one experiment to the other. That said, the fact that the findings of Experiment 2 were closely related to the findings of Experiment 1 speaks to the strength and validity of the current methodology to induce and measure cognitive fatigue in individuals with MS. Additionally, information on the physical disability level of the current subjects was unavailable, and could have been used to provide additional information on how cognitive fatigue and physical disability interact. Replication of these findings with larger sample sizes will be an important next step. However, even with the small sample, the current study showed that fMRI can be used to measure changes in the brain associated with fatigue. 

The current study utilized primarily two neuroimaging approaches (fMRI, DTI) to examine two aspects of cognitive fatigue (“state”, “trait”). Despite differences in neuroimaging modality and type of fatigue studied, the findings of experiment 1 and 2 converged to illustrate the importance of the role of a striato-thalamic-frontal cortical system in fatigue. The identification of a network of fatigue-related brain regions could be instrumental to reframe the current construct of cognitive fatigue and beginning to identify the pathophysiologic underpinnings of this multifaceted yet elusive symptom of MS.
